# Thoracolumbar Fracture in Disseminated Idiopathic Skeletal Hyperostosis

**DOI:** 10.7759/cureus.15222

**Published:** 2021-05-25

**Authors:** Lim Chia Hua, Sabarul A Mokhtar

**Affiliations:** 1 Department of Orthopedics and Traumatology, Faculty of Medicine, Universiti Kebangsaan Malaysia, Hospital Canselor Tuanku Muhriz, Kuala Lumpur, MYS

**Keywords:** dish fracture, ankylosing spinal disorder, osteoporosis, disseminated idiopathic skeletal hyperostosis, thoracolumbar fracture

## Abstract

Disseminated idiopathic skeletal hyperostosis (DISH) is a form of ankylosing spinal disorders, which is at high risk of fracture because of the rigidity of the spinal column and reduced bone quality. The patients with DISH are at higher risk of fall because of the poor muscle tone, rigid spine column, and positive sagittal balance. The management of spinal fractures in these patients proves to be challenging because of the altered biomechanics and alignment of the spine. Furthermore, most patients have multiple comorbidities with high intraoperative burden, and osteoporosis itself will impair any implant purchase of the bone. Here, we report a case of thoracolumbar fracture in DISH where both conservative and surgical approaches were utilized, with unfortunate results in both, and a brief review of the literature on its management.

## Introduction

Ankylosing spinal disorders comprise mainly ankylosing spondylitis (AS) and disseminated idiopathic skeletal hyperostosis (DISH). Another uncommon cause is the end-stage advanced spondylosis multiforme (EASM), all of which will end with rigid and often a deformed spine. These pathologies are also associated with poor bone quality. This makes the spinal column highly susceptible to fracture with trivial trauma with unfavorable outcomes. The average age was 63.4 years old and is more common in men with hyperextension injuries [1]. Usually, cervical spine fractures were more common than thoracolumbar, with the highest prevalence between C5 and C7 vertebra [1]. The management of an ankylosed spinal column fracture is challenging especially achieving stable fixation. Here, we reported a case of a thoracolumbar fracture in DISH with a brief review of the literature on its management.

## Case presentation

**Figure 1:**
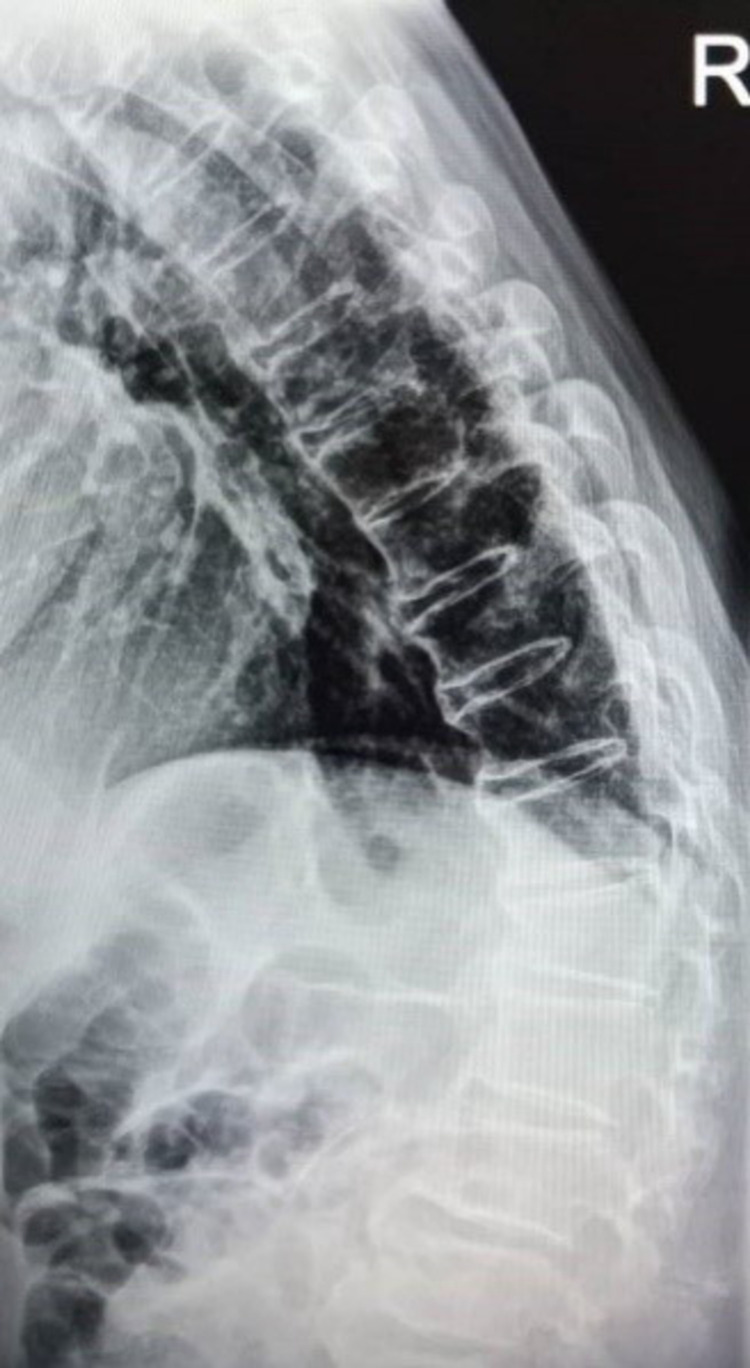
L1 compression fracture with anterior wedging <50%

**Figure 2:**
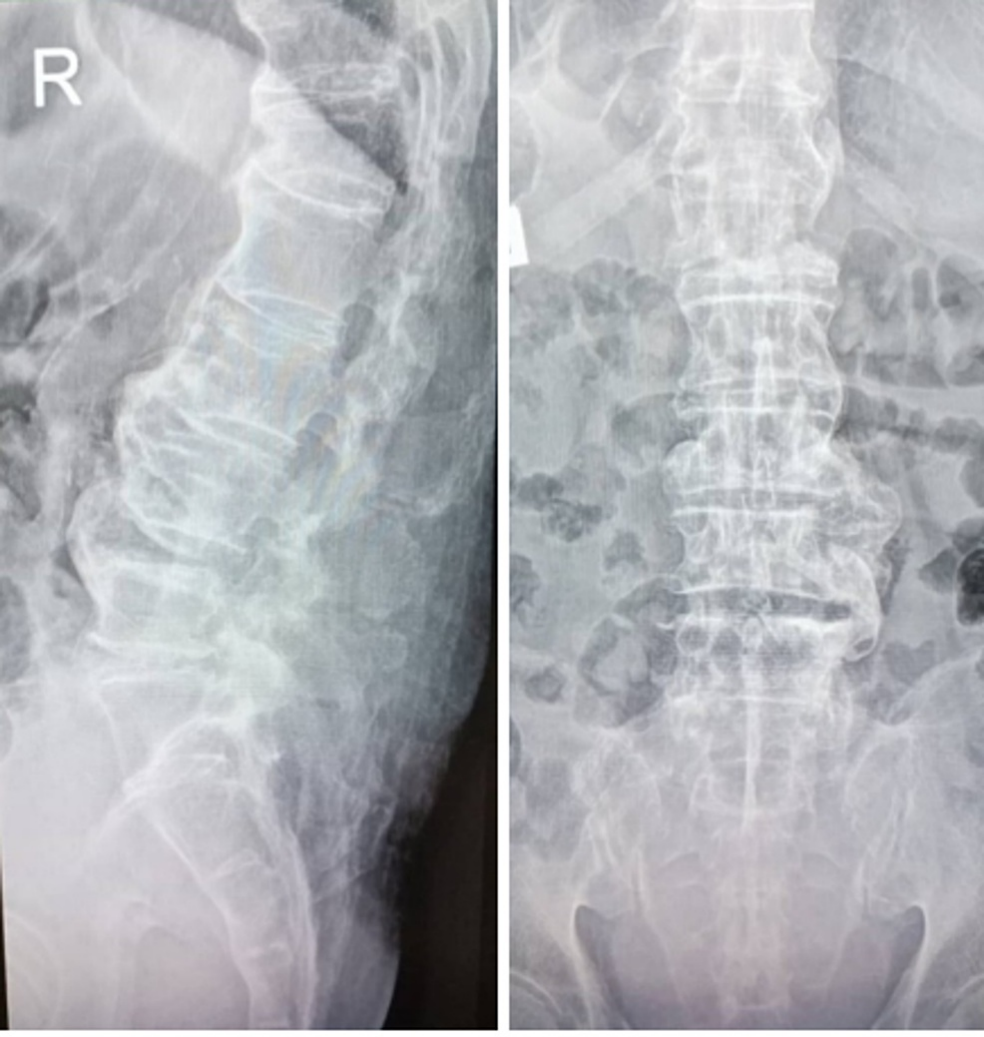
L1 compression fracture in DISH Disseminated idiopathic skeletal hyperostosis (DISH)

A 75-year-old man with underlying multiple comorbid hypertension, diabetes mellitus, dyslipidemia, bilateral eye cataract, and atrial fibrillation on warfarin was presented to us with a history of fall for which he sustained a closed fracture surgical neck of the left humerus and an L1 vertebra body compression fracture with anterior wedging less than 50% with an intact neurological function (Figure 1). There is no retropulsion bony fragment, and both the pedicles and posterior elements are intact. There is marginal ossification noted at the anterior and posterior aspects of the thoracolumbar vertebral bodies, but there was no sclerosis of bilateral sacroiliac joints, suggestive of underlying DISH (Figure 2).

He was treated conservatively for both his spine and humerus fractures in view of minimal fracture displacement and due to his underlying comorbidities. He was put on U-slab and Jewett brace for both his humerus and spine fractures, respectively. We recommended an anabolic agent as the best option of an anti-osteoporotic agent however he could only afford bisphosphonate. Otherwise, all blood parameters are normal, including inflammatory markers of C-reactive protein (CRP) and erythrocyte sedimentation rate (ESR), renal profile, and calcium levels. His bone mineral density test was normal for his age group. He was ambulating with a wheelchair on Jewett brace in the ward before he was discharged home. 

**Figure 3:**
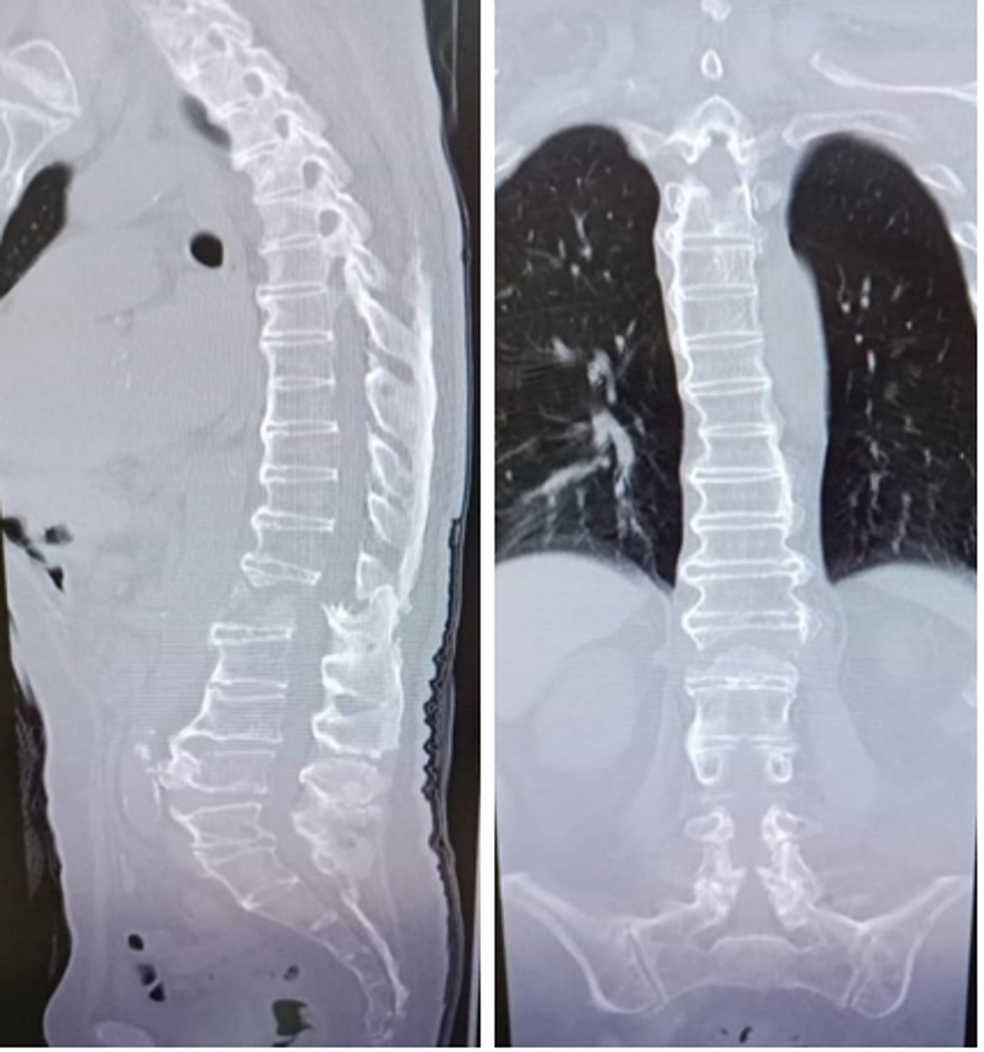
CT scan showing the L1 Chance fracture

**Figure 4:**
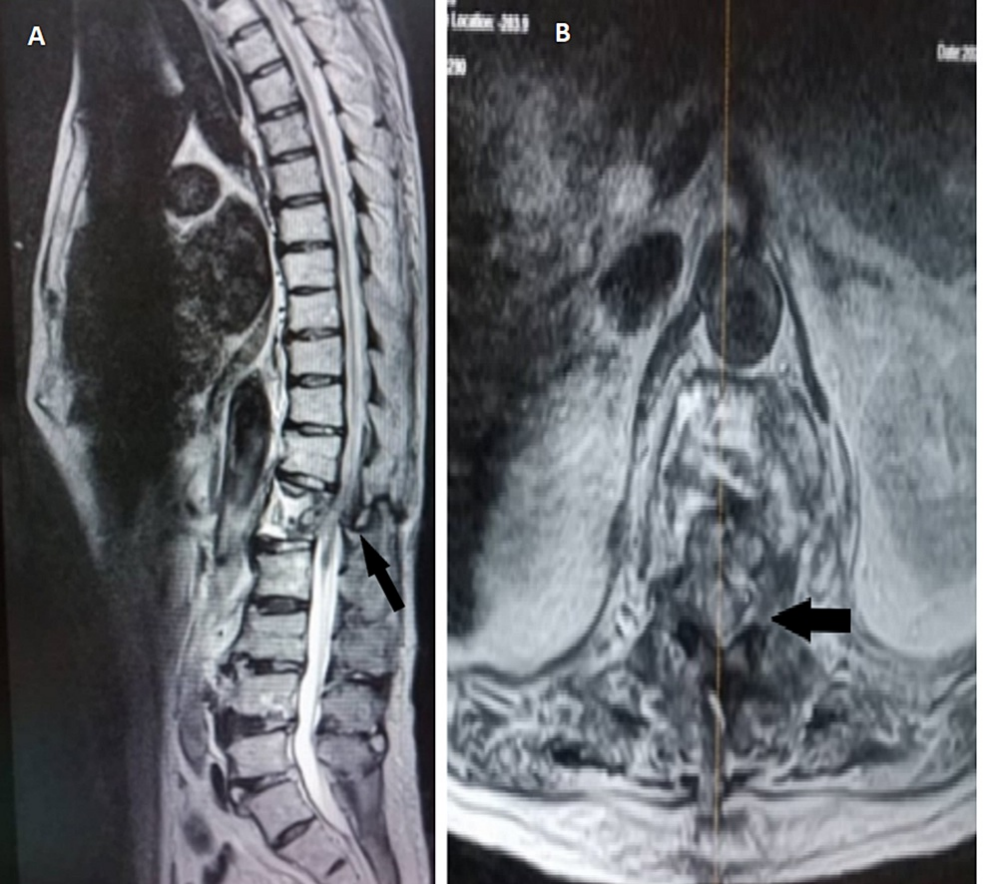
MRI showing intradural hematoma causing spinal canal stenosis and cord edema (black arrow)

A week later, he was readmitted with another fall due to imbalance; for this episode, he developed a worsening bilateral lower limb weakness which progresses to paresis, as well as bowel and bladder incontinence and saddle anesthesia. He developed a cauda equina syndrome with hematoma in the spinal canal. He was on warfarin for his atrial fibrillation, and his international normalized ratio (INR) was prolonged to more than six, which could be the cause of the intradural hematoma. This time, he sustained an L1 Chance fracture (Figure 3) with retropulsion displacement into the spinal canal and intradural hematoma (Figure 4A), causing significant spinal canal stenosis with spinal cord edema (Figure 4B).

**Figure 5:**
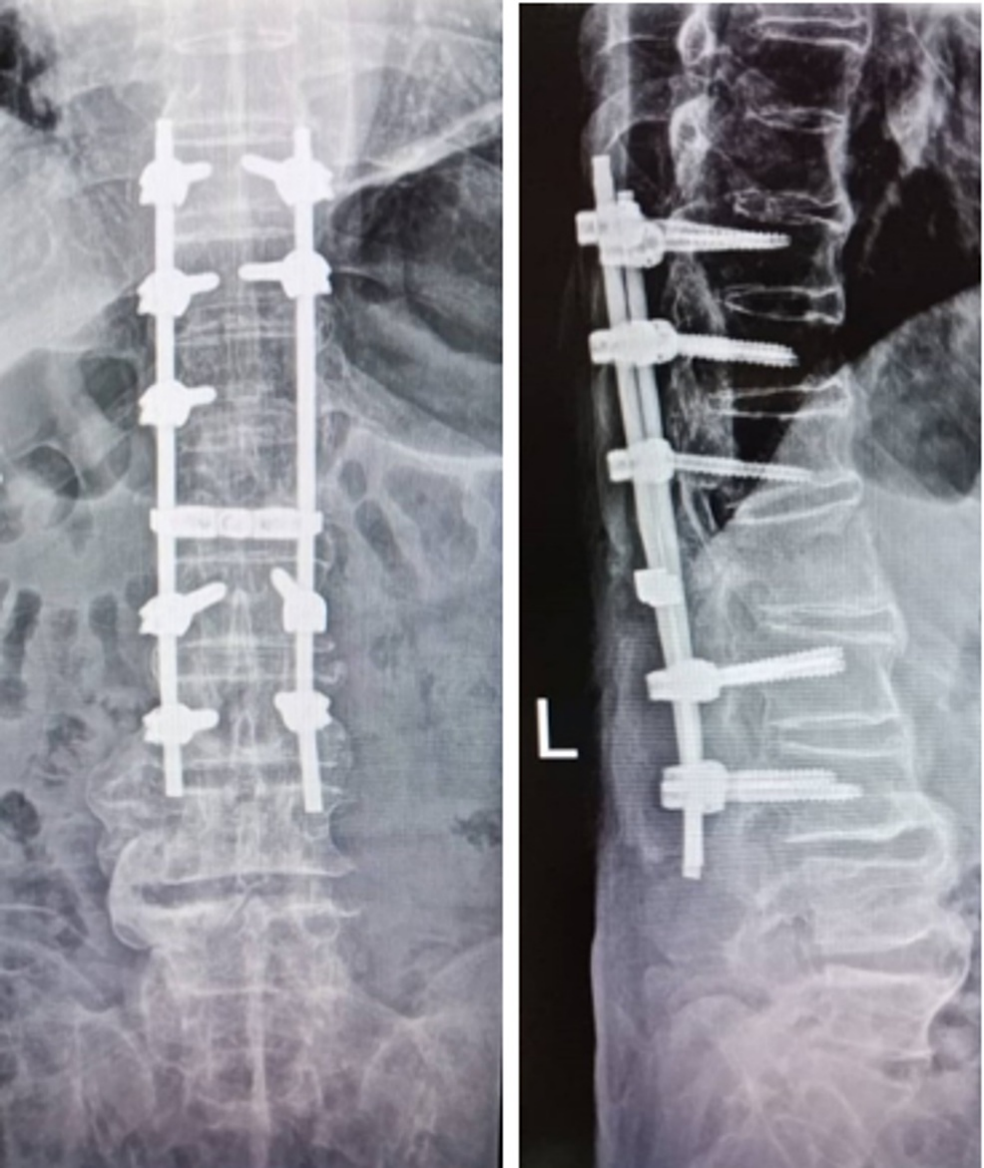
Post spinal stabilization and decompression

Upon further questioning, he has been non-compliant with his brace at home and was ambulating without the brace. Spinal stabilization and decompression surgery were done for him with three levels above and two levels below the fracture after the INR was normalized (Figure 5). 

He had no complications during surgery, but after two weeks, he developed pneumonia and wound breakdown over the surgical site at the spine with methicillin-resistant Staphylococcus aureus (MRSA) bacteremia. He underwent multiple debridements for his surgical site infection over the spine. Subsequently, he developed multi-organ failure six months later and succumbed to sepsis.

## Discussion

Disseminated idiopathic skeletal hyperostosis (DISH), also known as Forestier disease, is a noninflammatory disease that is more common in males of advanced age and patients with multiple comorbid of pulmonary hypertension, diabetes mellitus, metabolic syndrome, and obesity [1]. Causes remain unknown mainly, and the association between DISH and HLA-B27 has been inconclusive [2]. DISH typically manifests in the thoracic spine (T7-T11), with typically more than three segments coalesced into hypertrophic exophytic bony overgrowth seen in the anterior longitudinal ligament with minimal involvement of paravertebral connective tissue. The ossification process may involve more than one spinal region, usually extends laterally along the anterior longitudinal ligament (ALL) with non-marginal syndesmophytes. The next most commonly affected spine is the lumbar, followed by the cervical spine. If ossification extends and involves the ligamentum flavum in the lumbar spine and posterior longitudinal spine in the cervical, it might result in spinal stenosis. The key differences between AS and DISH are the absence of sacroiliac joint involvement, no syndesmophyte formation, and absence of craniocervical joints involvement radiologically in DISH. Clinically, DISH is more at risk of the neurological deficit because of high-grade spinal stenosis [3]. 

Studies have shown that patients with AS or DISH are at a higher risk of fall and spine fractures because of the poor muscle tone, rigid spine column, and positive sagittal balance. Complete cord injury is seen more commonly with a rigid spine, and spinal cord injury is a disastrous complication of spinal fracture in these patients. The rate of spinal cord injury after traumatic spine fracture ranges between 19% and 97% in AS and DISH [4].

Osteoporosis is one of the important factors in AS or DISH, especially in the evolution of spontaneous vertebra fractures in the spine, which poses problems in surgery. Poor mineralization of bone results from the ectopic bone being formed in ongoing inflammation areas with high osteoclastic activity. Measuring bone mineral density (BMD) with conventional dual-energy X-ray absorptiometry (DEXA) scans usually gives false-positive rates in the presence of syndesmophytes. Our patient has a normal BMD result according to his age.

The management of spinal fractures in these patients proves to be challenging because of the altered biomechanics and alignment of the spine. Furthermore, most patients have multiple comorbidities with high intraoperative burden, and osteoporosis itself will impair any implant purchase of the bone. Generally, nondisplaced and clinically stable spine fractures can be treated nonsurgically with bracing in the thoracolumbar fractures. However, a high failure rate of almost 50% with secondary fracture displacement was reported in patients who are not compliant with bracing [5]. Our patient was treated conservatively with a Jewett brace initially due to undisplaced fracture and intact neurological function. He was discharged well with advice on both the importance of compliance with the Jewett brace and osteoporosis medication. However, he returned to us a week later with another fall and, this time, a displaced fracture with a complete neurological deficit. The further assessment shows that he has not been compliant with his brace at home. At this point, the only option is a surgical intervention with decompression and stabilization surgery.

Surgical intervention is recommended in the presence of neurological deterioration, unstable fracture, and epidural hematoma. Whang et al. compare the treatment options and reported a good outcome of 85% in patients treated surgically [6]. Caron et al. reported a mortality rate at one year to be 51% in non-surgical treated patients compared to 32% in surgically treated patients [7]. Lu et al. reported reversal of neurological deficit and solid fusion after surgical treatment [8]. At the same time, Westerveld et al. further confirmed no further neurological decline in 59% of cases and improvement in 27% of cases in surgically treated patients [4,9]. Werner et al. recommended a long posterior stabilizing spine surgery with three levels above and three levels below the fracture level [5]. The technical challenges would be the pedicle screw placement in severely distorted facet joints in AS or DISH and osteoporotic bone.

For patients with deformity of the spine, optimal spinal stabilization with or without deformity correction remains controversial. Complications with operative stabilization and fusion such as pneumonia, respiratory failure, wound breakdown, and pseudoarthrosis were seen in up to 84% of patients with mortality rate ranged from 0% to 32% at one year postinjury [1]. As shown in our case, we had tried both the conservative and surgical approaches. Conservative will only be successful with the patient’s compliance to bracing; perhaps better communication with the patient and his caretaker would be helpful. Although the surgical approach shows promising results in the literature, the complications postoperatively were devastating.

## Conclusions

In conclusion, DISH is a form of ankylosing spinal disorder, which is at a high risk of fracture because of the rigidity of the spinal column and reduced bone quality. Although surgical stabilization will be an ideal treatment for vertebral fracture with DISH, conservative treatment should be considered in cases with high operative risk. For conservative, the patient's compliance to brace must be emphasized. Surgical management of an ankylosing spinal fracture, on the other hand, is challenging due to the pre-existing spinal deformity along with a rigid yet brittle spinal column. All factors must be considered when choosing the best treatment for patients.
